# An exploratory method to determine the plant characteristics affecting the final yield of *Echium amoenum* Fisch. & C.A. Mey. under fertilizers application and plant densities

**DOI:** 10.1038/s41598-022-05724-8

**Published:** 2022-02-03

**Authors:** Mohammad B. Amiri, Mohsen Jahan, Parviz Rezvani Moghaddam

**Affiliations:** 1 Department of Agronomy and Plant Production, University of Gonabad, Gonabad, Iran; 2 Department of Agrotechnology, Faculty of Agriculture, Ferdowsi University of Mashhad (FUM), Azadi Sq., 9177948978 Mashhad, Iran

**Keywords:** Plant sciences, Environmental sciences

## Abstract

Employing of advanced statistical methods to quantify agricultural information has helped to carry out targeted planning to alleviate the problems of farmers, researchers and policy section. One of these exploratory methods, is multivariate statistical analysis that examines and models the relationship between variables. Considering the importance of *Echium amoenum* and its use growing trend in traditional medicine and the pharmaceutical industry, also the lack of information on the correlations between its yield and morpho physiological traits, the objective of this study was to determine the causality path in which the *Echium amoenum* characteristics affects the yield of *Echium amoenum* as regards of application of organic and chemical fertilizers under different plant densities. The employed method revealed that organic fertilizers increased flower yield compared with the control. The flower yield as a result of application of compost, vermicompost and cattle manure were increased by 25, 28, and 27% compared with the control, respectively. The results of multiple regression showed that variables of plant height, shoot dry weight, flower number per plant were the main factors affected the flower yield. The relative contribution of shoot dry weight was 16 and 25% more than plant height and flower number per plant, respectively. Causality analysis identified that shoot dry weight per plant had indirect effect on flower yield in different paths, as mainly was imposed through plant height considering the path coefficients. This study suggests that optimum production of *Echium amoenum* with application of ecological inputs along with effective agronomical managements of the causal paths of flower yield forming, including increase in shoot yield and plant height could be achieved through an ecological cropping system with reduced costs and no health concerning due to agrochemicals residual.

## Introduction

Currently, the use of eco-friendly inputs as approaches to achieve sustainable agriculture has been considered^1,2^. Undoubtedly, the application of organic fertilizers and manures especially in nutrient-poor soils in addition to its positive effects on all soil properties and the increase of its organic matter, can also be beneficial in economic, environmental and social aspects and can be a suitable and desirable substitute for chemical fertilizers in long-term^3,4^. Application of animal manure increases soil yield, improves water holding capacity and improves soil aggregation and increases water use efficiency and increases crop yield^5^. Organic compost and vermicompost in most parts of the world have been successfully used on a large number of agricultural products^6,7^. It was reported that the highest root growth rate and root relative growth rate of sorghum (*Sorghum bicolor* L.) were resulted from the combined application of vermicompost and mycorrhiza treatment^8^. Introducing these organic fertilizers to soil improves nutritional, physical, chemical and biological aspects of soil ecosystem are also improved^9,10^^,^^11^. The result of an experiment revealed that combined application of the biofertilizer mixture (*Azotobacter chrocoocum*, AMF, and *Bacillus circulans*) with organic fertilizers enhanced maize growth, yield, and nutrient uptake. Moreover, the bio-organic fertilization has improved the soluble sugars, starch, carbohydrates, protein, and amino acid contents in maize seeds. Additionally, the bio-organic fertilization caused an obvious increase in the microbial activity by enhancing acid phosphatase and dehydrogenase enzymes, bacterial count, and mycorrhizal colonization levels in maize rhizosphere as compared with the chemical fertilization^12^. It was reported that the application of biofertilizers including *Bradyrhizobium* sp., *Bacillus subtilis*, and arbuscular mycorrhizal fungi (AMF), individually or in combinations, improved Guar (*Cyamopsis tetragonoloba)* shoot length, root length, number of branches, plant dry weight, leaf area index (LAI), chlorophyll content, and nutrient uptake of guar plants compared with the control plants. Moreover, the application with biofertilizers resulted in an obvious increase in seed yield and has improved the total proteins, carbohydrates, fats, starch, and guaran contents in the seeds. Additionally, biofertilizer treatments have improved the soil microbial activity by increasing dehydrogenase, phosphatase, protease, and invertase enzymes. Soil inoculation with the optimized doses of biofertilizers saved about 25% of the chemical fertilizers required for the entire guar growth stages^13^. In a study the effect of different organic fertilizers on the quantitative and qualitative properties of several medicinal plants was investigated, it was reported that vermicompost increased *Echinaceae purpurea* L. plant height and increased fresh and dry shoot weight of *Melissa officinalis* L^14^. It was reported that the application of organic fertilizers, especially vermicompost, on *Calendula officinalis* L. resulted in a significant increase in the number of branches per plant and the flower number per plant^15^. In another study, the application of vermicompost increased dry matter of tomato (*Lycopersicum esculentum* L.) also improved nitrogen uptake efficiency in organic tomato production^16^. It has been reported that single or co-inoculation of soybean by *Bacillus amyloliquefaciens* and/or Arbuscular Mycorrhizal Fungi (AMF) as natural biofertilizers strengthen the positive effect of drought on the antioxidant and osmoprotectant levels, i.e., phenol, flavonoid, glycine betaine contents, and glutathione-S-transferase (GST) activity. As a result of stress release, there was a decrease in the level of stress hormones (abscisic acid, ABA) and an increase in gibberellin (GA), trans-zeatin-riboside (ZR), and indole acetic acid (IAA) in the seeds of inoculated plants^17^.

Plant density, as an agronomical management practice, plays an important role in the yield of different crops, so identifying the optimum plant density is one of the basic principles of crop production^18^. A balanced increase in plant density will accelerate canopy closure, increase leaf area, increase productivity of environmental resources, reduce weeds and ultimately improve the yield and yield components of different plants^19,20^. It is remarkable that at high plant densities, leaf loss rate increased and consequently. It has negative effects on the quantitative and qualitative characteristics of the plant, due to shading and competition of plants for light and scarcity of available resources, as well as greater susceptibility of plants to pathogens^21^. In a study, the effect of different plant densities (12.5, 16.6 and 25 plants m^−2^) on yield and yield components of Coriander (*Coriandrum sativum* L.) was studied and reported that, the number of umbrellas per plant, 1000-seed weight and plant dry weight decreased with increasing density^22^. In another experiment, the effect of different plant densities on yield and yield components of a medicinal plant (*Hibiscus sabdariffa* L.) was studied and reported that with increasing the distance between planting rows from 50 to 100 cm, flower yield increased^23^.

In recent years, the use of advanced statistical methods to quantify agricultural information has helped to carry out targeted planning to alleviate the problems of farmers, researchers and policy section. One of these methods, which is widely used in all sciences disciplines today, particularly agricultural sciences, is multivariate statistical analysis that examines and models the relationship between variables^24,25^. One of the exploratory multivariate methods as an elaborating statistical method in analyzing and explaining many phenomena is causality analysis or path analysis method. Causality analysis is a precise analytical tool to determine the share of direct and indirect effects of one variable with the other ones, since many studies have found that one variable not only has a direct effect on the other variable, but also indirectly through other variables affects those^26^. In other words, causality analysis divides the correlation coefficient between the two variables into a direct and indirect effects^27,28^. In a study, the relationships between yield and yield components of spring safflower (*Carthamus tinctorius* L.) were investigated and reported that biological yield, number of pod and branch and number of grains per pod were effective on grain yield, also according to the results of stepwise regression, whereas path analysis results showed that only two of the four traits (biological yield and number of pods per plant) effectively affected grain yield^29^.

*Echium amoenum* Fisch. & Mey. is a perennial herb, Boraginaceae family plant, and is a valuable herb due to excellent medicinal properties^30^. This plant has been distributed across the northern parts of the country as wild vegetation^31^. In traditional medicine, the petals of this plant are used as diuretics, analgesics, diaphoretic, and treat for high blood pressure^32,33^. Considering the importance of *Echium amoenum* and its use growing trend in traditional medicine and the pharmaceutical industry, also the lack of information on the correlations between its yield and morpho physiological traits, this study was conducted aimed to determine the causality path in which the borage characteristics affects the yield of *Echium amoenum* as regards of application of organic and chemical fertilizers under different plant densities.

## Materials and methods

### Site description

Field studies were conducted during the 2013–2014, 2014–2015 and 2015–2016 growing seasons at the Research Farm Station of Agriculture Faculty, Ferdowsi University of Mashhad, Iran (latitude: 36° 15 N; longitude: 59° 28 E; elevation: 985 m above sea level). The Research station was located in Kashaf-rood watershed in northeast of the country in a semi-arid region with mean annual precipitation of 252 mm and temperature of 15° C. Documented declaration of cropping history of the land which experiment was conducted in confirmed that it had been under fallow for the past three years, with no agrochemicals chemicals consumed or imported in (Research Station Archive).

Soil samples were taken at 0–30 cm depths and analyzed for some physiochemical properties^34^ before conducting the experiment (Table 1).

**Table 1:** Soil properties of the experimental field.

Soil texture	Total nitrogen (ppm)	Available phosphorous (ppm)	Available potassium (ppm)	pH	EC (dS m^−1^)
Silty loam	15.7	13.4	417	7.3	1.1

### Experimental design

A split plot arrangement based on a RCBD design with three replications was conducted. Three plant densities (10, 5 and 3 plant m^−2^) were assigned to the main plots and five different types of organic and chemical fertilizers (compost by 10 t ha^−1^, vermicompost by 7 t ha^−1^, cattle manure by 30 t ha^−1^, chemical nitrogen fertilizer as Urea by 180 kg ha^−1^ and the control) were assigned to the sub plots. Since *Echium amoenum* is a perennial plant, the experimental blocks and plots containing the underground parts and crown of borage plants of the first growing season were reserved intact for the second and third cropping year.

### Soil and treatments preparation and crop management

Minimum tillage was carried out to prepare the soil with emphasis on ecological soil cultivation operations, so that after a shallow disk, plots of 2.5 × 5 m with a distance of 1 m between, to avoid nutrients mixing due to irrigation consisting of 6 rows were arranged to sow the borage seeds on the middle of rows.

To applying organic fertilizers, the amounts of NPK in compost, vermicompost and cattle manure were determined (the results of the analysis of organic fertilizers used in the experiment shown in Table 2), then according to NPK requirements of *Echium amoenum*^35^ as well as taking into account the local farmers recommendations, the needed amounts of fertilizers were determined. Pure nitrogen by 90 kg ha^−1^ (this amount of pure nitrogen was provided by 180 kg urea fertilizer containing 46% N), half of which at the time of sowing and the other half after thinning operation were applied, while in the second cropping year (2014–2015), the same amount of fertilizer was added in two stages (beginning of regrowth and four-leaves stages in the second year).

**Table 2:** Chemical analysis results of organic fertilizers used in the experiment.

Type of organic fertilizer	Nitrogen (%)	Phosphorous (%)	Potassium (%)
Compost	0.64	0.44	0.49
Vermicompost	0.89	1.53	0.96
Cattle manure	0.21	0.29	1.04

In late February 2013, organic fertilizers were broadcasted on the soil surface uniformly and immediately were mixed into the soil (a depth of 30 cm) of related plots using a spade. In late February 2014, to promote the plants regrowth, the same amount of fertilizers were added into the soil on the side of planting rows of the related plots in a depth of 15 cm.

Seeds were sown on April 5, 2013. Replanting was done after seed emergence where needed. Plots were immediately irrigated after sowing and later at 7-days interval. After reaching the plant to a 4-leaves stage, thinning was carried out to reach the appropriate density. Borage plants were established in the first year and no sampling or measurement was done during this growing season (2012–2013). The data of this study are recorded from the second and third year of experiment (2013–2014, 2014–2015).

To control weeds, weeding was done three times in the first year (15, 30 and 45 days after planting, respectively) and (30 days after plant regrowth in second and third years. No herbicides, pesticides and chemical fungicides were used to prepare the soil during the growing seasons in three years of the study.

### Plant sampling and measurements

At the spring of the second and third years (2015–2016) during of the flowering season (April 6, to June 20) the flowers of all experimental plots were harvested daily (borage is identified with an undetermined growth pattern) then fresh and dry weights of flowers were measured. Harvested flowers were air dried under the shadow avoiding direct sunlight. The total dry weight of flowers during the flowering period was considered as the dry flower yield per plot. Three plants per plot were randomly selected and the flower number were counted during the flowering period.

At the end of the growing season, with the onset of seed ripening and plant shoot drying, three plants were randomly selected from each plot and traits including shoot yield, branch number, branch length, plant height and their canopy diameters were measured. To determine seed yield, total plants of all experimental plots were harvested and seed weight was determined.

### Data statistical analysis

A normality test was already performed. Transformation was also performed for numerical data where needed. To ensure uniformity of treatment variances, the Bartlett's test was performed. Since there was no statistical difference between experiment data of two years (2014–2015, 2015–2016), thus the mean of each trait values during two years were reported. Analysis of variance (ANOVA) and graph plotting were done using SAS Ver.9.1, Slide Write Ver.2 and Microsoft Excel Ver. 14. All mean comparisons were performed by Duncan's multiple range test (DMRT) at 5% probability level. Growth characteristics affecting dry flower yield were determined using multiple regression and Minitab Ver.16 software. In order to find out the causal relationships between yield and growth characteristics affecting it, causality analysis was performed^28^.

### Ethics approval and consent to participate

This research meets all the ethical guidelines, including adherence to the legal requirements of my country.

### Consent for publication 

The authors confirm no conflict of interest and agree with the submission of the manuscript to Scientific Reports journal.

## Research and publication ethics

The authors confirm that the use of plants in the present study complies with international, national and institutional guidelines.

## Results

### Shoot yield

The effect of plant density on shoot yield was significant (Table 3), as the highest shoot yield per plant resulted from medium density (5 plant m^−2^). This plant density increased yield by 34 and 47%, compared with densities of 10 and 3 plant m^−2^, respectively. Different organic and chemical fertilizers had a significant effect on shoot yield (Table 3). All the organic fertilizers had a positive effect on shoot yield as shoot yield as a result of application of compost, vermicompost and cattle manure increased by 25, 7 and 19%, respectively, compared with the control. The chemical fertilizer also resulted in 17% increase in shoot yield compared with the control. Compost and cattle manure increased shoot yield by 10% and 2%, respectively compared with chemical fertilizer.

**Table 3:** Analysis of variance (mean of squares) of some growth characteristics and yield of *Echium amoenum* affected by different types of fertilizers and plant densities.

Source of variation	d.f	Mean of squares			
		Shoot yield per plant	Plant height	Flower number per plant	Dry flower yield
Block	2	8.80^ns^	109.95^ns^	221,078^ns^	339,294^ns^
Plant density	2	48,116.71**	0.82^ns^	2,810,317**	13,075,569**
Fertilizer	4	3396.94**	540.38**	1,722,380**	2,183,909**
Plant density × fertilizer	8	9373.31**	122.40^ns^	923,445**	2,497,218**
Experimental error	28	305.51	74.91	191,739	266,634
CV (%)	–	10.36	9.48	17.58	16.84

**, * and ns are significant at the 0.01 and 0.05 of probability level and non-significant, respectively

Interaction effects of plant density and organic and chemical fertilizers on shoot yield showed that the highest (312.15 g per plant) and the lowest shoot yield (61.28 g per plant) were obtained from treatments of 5 plants m^−2^ plus compost fertilizer and 3.3 plant m^−2^ plus control, respectively (Table 4).

**Table 4:** Mean comparisons of interaction of different densities and organic and chemical fertilizers application on some characteristics and yield of *Echium amoenum.*

	Type of fertilizer	Shoot yield per plant (g plant^−1^)	Plant height (cm)	Flower number per plant	Dry flower yield (kg ha^−1^)
Density of 3 plants per m^−2^	Compost	151.00ef	99.00ab	2268.1ed	3914.4ab
	Vermicompost	96.12h	86.33a–c	2594.9b–d	4340.7a
	Cattle manure	126.33f–h	90.00a-c	3348.3b	2996.6bc
	Chemical fertilizer	170.98de	101.66a	2489.8cd	4392.1a
	Control	210.01c	78.00c	2528.9b-d	4219.3a
Density of 5 plants per m^−2^	Compost	312.15a	101.33a	2178.3	3654.0ab
	Vermicompost	262.27b	96.00ab	2535.7b-d	4242.9a
	Cattle manure	217.65c	98.66ab	4177.7a	3580.2ab
	Chemical fertilizer	207.19c	82.33bc	2576.3b-d	2354.4c
	Control	161.14de	77.66c	2650.4b-d	2142.7c
Density of 10 plants per m^−2^	Compost	116.84gh	100.00a	1844.6de	2504.4c
	Vermicompost	105.58 h	90.00a-c	3123.7bc	2117.3c
	Cattle manure	188.40 cd	98.66ab	1871.6de	3818.6ab
	Chemical fertilizer	143.30e-g	86.00a-c	1640.9e	913.8d
	Control	61.28i	82.66bc	1524.6e	1183.4d

In each column, means followed by the same letters are not significantly different (*p* ≤ 0.05), based on Duncan’s multiple range test.

With decreasing of density, the trend of shoot yield changes in different organic fertilizers was similar, so that under all organic fertilizers application condition, by decreasing densities down to 5 and 3 plants m^−2^, shoots yield was increased first and then decreased (Table 4). As it shown in Table 4, chemical fertilizer in 5 and 3 plant m^−2^ increased shoot yield by 22 and 62%, respectively compared with the control.

### Plant height

Although the effect of different densities on plant height was not significant, plant height was affected by different organic and chemical fertilizers (Table 3), so that all organic fertilizers increased plant height compared with the control. Application of compost, vermicompost and cattle manure resulted in increased plant height by 21, 12 and 17%, respectively, while the average plant height under these fertilizer applications was more than the control by 12%. All of the organic fertilizers had superiority to chemical fertilizer regarding plant height, so the plant height as a result of application of compost, vermicompost and cattle manure were higher than chemical fertilizer by 10, 4 and 6%, respectively.

As it shown in Table 4, under all plant densities, organic fertilizers increased plant height compared to the control, as in density of 10 plant m^−2^, the plant height was higher under application of compost, vermicompost and cattle manure by 21, 10 and 13% respectively. In density of 5 plant m^−2^, these amounts were by 23, 19 and 21% and in density of 3 plant m^−2^ plant high were higher than the control by 17, 8 and 16% respectively.

### Flower number per plant

Plant density had a significant effect on the flower number per plant (Table 3), as with increasing plant density up to 5 plants m^−2^, the flower number per plant was increased, while increasing the density (up to 10 plants m^−2^) decreased the flower number per plant.

There was a significant difference between different organic and chemical fertilizers as regards the effect on flower number per plant (Table 3), as vermicompost and cattle manure increased this trait by 19 and 29%, respectively, compared with the control. Application of these organic fertilizers also resulted in increased flower number per plant compared with the chemical fertilizer.

The interaction of the effect of organic fertilizers was different at different plant densities, as cattle manure in densities of 10 and 5 plants m^−2^, vermicompost in 3 plants m^−2^ were significantly higher than the other treatments. Compost, vermicompost, and cattle performing their best in increasing flower number per plant in densities of 10, 3 and 5 plants per m^−2^ respectively (Table 4).

As it shown in Table 4, fertilizer in all densities had no significant effect on flower number per plant compared with control, but it seems that at 5 plant m^−2^ had effective impact on plant density as its application in 5 plants m^−2^ resulted in increased flower number per plant by 3 and 36%, respectively, compared with application of fertilizer in 10 and 3 plant densities, respectively.

### Dry flower yield

Dry flower yield was significantly affected by plant density (Table 3), as the highest flower yield (3972.6 kg ha^−1^) was obtained from density of 10 plants m^−2^ which was higher than densities of 5 and 3 plants m^−2^ by 21 and 47%, respectively. The effect of organic and chemical fertilizers on dry flower yield was significant (Table 3), as all organic fertilizers increased dry flower yield compared with control. Dry flower yield resulted from application of compost, vermicompost and cattle manure were higher by 25, 28 and 27% than the control, respectively. It is remarkable that all organic fertilizers (compost, vermicompost and cattle manure) had higher dry flower yield than chemical fertilizer by 24, 27 and 26%, respectively.

The interaction of plant density and fertilizers on dry flower yield was significant (Table 3), as by decreasing plant density, the efficiency of organic fertilizers in increasing dry flower yield was increased. Organic fertilizers had no significant effect on dry flower yield in density of 10 plants m^−2^, whereas compost, vermicompost and cattle manure increased dry flower yields by 41, 49 and 40%, respectively compared with the control. In density of 3 plants m^−2^ application of compost, vermicompost and cattle manure to soil increased dry flower yield by 53, 44 and 69%, respectively compared with the control (Table 4).

As it shown in Table 4, the effect of organic fertilizers was different at different among plant densities, as in densities of 10 and 5 plants m^−2^ application of vermicompost, and in density of 3 plants m^−2^ cattle manure application resulted in more increased dry flower yield than other treatments.

### Relative comparison of growth characteristics of borage under plant densities

**Figure 1:**
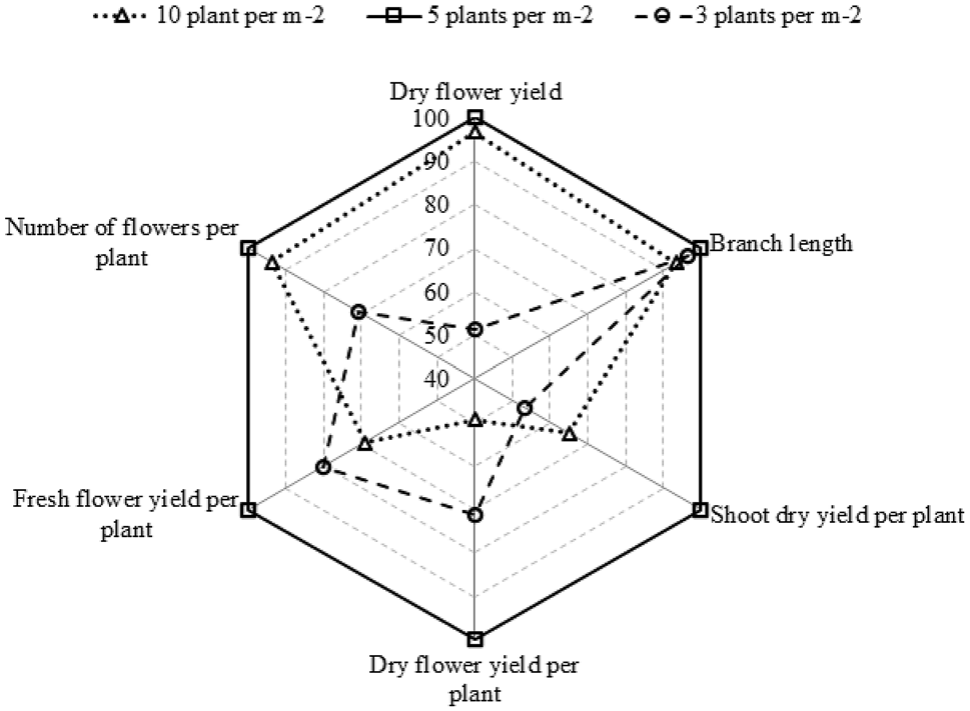
Relative comparison of *Echium amoenum* growth characteristics under three plant densities: 5 plants per m^−2^ (continuous line), 10 plants per m^−2^ (dotted line), and 3 plants per m^−2^ (cut line).

Relative values of growth characteristics of borage under different densities shown in Fig. 1.

The advantage of the density 5 plant per m^−2^ considering fresh flower yield, shoot dry yield, dry flower yield is clearly revealed.

### Coefficients of correlation between traits

As shown in Table 5, the number of branches per plant was the only trait that had a significant positive correlation with the flower number per plant. Correlations between fresh and dry weight of flowers per plant with all studied traits were significant except of flower number per plant and canopy diameter. Seed yield was also correlated with most physio morphological traits. The shoot yield per plant was significantly correlated with fresh and dry flower weight per plant, seed yield and branch length (Table 5).

**Table 5:** Coefficients of correlation between growth characteristics and yield of *Echium amoenum* affected by different types of fertilizers and plant densities.

Code	Trait	1	2	3	4	5	6	7	8	9	10
1	Dry flower yield	1									
2	Flower number per plant	0.30*	1								
3	Fresh flower weight per plant	0.40**	0.10^ns^	1							
4	Dry flower weight per plant	0.36*	− 0.0^ns^	0.76**	1						
5	Seed yield	0.26^ns^	− 0.12^ns^	0.67**	0.67**	1					
6	Shoot yield per plant	0.42**	0.16^ns^	0.59**	0.47**	0.34*	1				
7	Branch number per plant	0.31*	0.30*	0.55**	0.56**	0.35*	0.22^ns^	1			
8	Branch length	0.28*	0.09^ns^	0.42**	0.41**	0.42**	0.35*	0.22^ns^	1		
9	Plant height	0.35*	0.03^ns^	0.41**	0.38**	0.34*	0.22^ns^	0.22^ns^	0.53**	1	
10	Canopy diameter	− 0.05^ns^	0.09^ns^	− 0.10^ns^	0.06^ns^	− 0.10	0.14^ns^	0.12^ns^	0.23^ns^	0.05^ns^	1

**, * and ns are significant at the 0.01 and 0.05 of probability level and non-significant, respectively

Although there was a significant correlation between the number of branches per plant and most of the studied traits, its correlation with fresh weight (r = 0.55**) and flower dry weight per plant (r = 0.56**) was more than the correlations of this trait with the other ones. Plant height and branch length were correlated with most of the studied traits, but the canopy diameter was not significantly correlated with any of the studied traits (Table 5).

The correlation between most studied morphological traits and dry flower yield was positive and significant, as the highest correlation was related to shoot yield per plant (r = 0.42**), flower fresh weight (r = 0.40**), and flower dry weight per plant (r = 0.36*), and plant height (r = 0.35*) (Table 5). The correlations of number of branches per plant, branch length and flower number per plant were also significant with dry flower yield, so by increasing each of them, dry flower yield would be improved (Table 5).

### Identified growth characteristics affecting dry flower yield using multiple regression

The results presented in Table 5 showed that the flower yield of *Echium amoenum* was correlated with most of the measured variables. Accordingly, multiple regression was used to analyze the relationship between flower yield as a function variable (Y) and traits affecting it (independent variables, X). For this purpose, first of all the variables which are studied including number of branches per plant (X1), branch length (X2), plant height (X3), canopy diameter (X4), seed yield (X5), shoot yield (X6), fresh flower weight per plant (X7), dry flower weight per plant (X8) and flower number per plant (X9) were included in regression model. At the first step of the regression, the relationship between flower yield (Y) and all the studied variables (X1, …, Xn) was estimated. The coefficient of determination of this model was calculated of *R*^2^ = 0.62**. Then, the backward stepwise regression method was performed to eliminate the variables having weak coefficient of determination in model. Regression results showed that variables of plant height (X3), shoot yield per plant (X6) and flower number per plant (X9) were the main factors affecting the flower yield of *Echium amoenum* (Eq. 1).

1 $$ {\text{Y }} = \, 0.0{11}0{4 } + \, \left( {0.{32 } \times {\text{ X6}}} \right) \, + \, \left( {0.{27 } \times {\text{ X3}}} \right) \, + \, \left( {0.{24 } \times {\text{ X9}}} \right) \,{\text{r }}\, = \, 0.{55}* $$

where Y = dry flower yield (kg ha^−1^), X6 = shoot yield per plant (g), X3 = plant height (cm), and X9 = flower number per plant.

### The causal paths of independent variables affect dry flower yield forming

**Figure 2:**
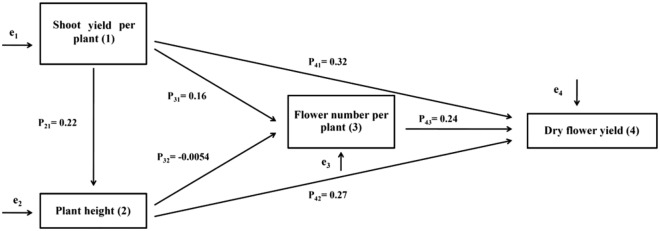
Path coefficients diagram showing the causality path of dry flower yield forming and growth characteristics of *Echium amoenum* as a result of application of organic and chemical fertilizers and different plant densities (e represents non-measurable errors).

After identifying the main growth characteristics affecting dry flower yield using multiple regression, the direct and indirect effects of each of these characteristics were estimated using causality (path) analysis method. The effect of these characteristics on each other and on dry flower yield has been shown in Fig. 2.

Equations 2, 3, 4, 5, 6 and 7were used to calculate the coefficients of direct effects of growth characteristics on each other and on dry flower yield shown in Fig. 2^28^:

2 $$ {\text{Direct}}\;{\text{ effect}}\;{\text{ of}}\;{\text{ shoot}}\;{\text{ weight }}\;{\text{per}}\;{\text{ plant }}\;{\text{on}}\;{\text{ the}}\;{\text{ plant }}\;{\text{height}}:{\text{P}}_{{{21}}} = {\text{ r}}_{{{12}}} $$

3 $$ {\text{Direct }}\;{\text{effect }}\;{\text{of }}\;{\text{shoot }}\;{\text{weight}}\;{\text{ per }}\;{\text{plant}}\;{\text{ on }}\;{\text{the}}\;{\text{ flower }}\;{\text{number}}\;{\text{ per }}\;{\text{plant}}:{\text{P}}_{{{31}}} = {\text{ r}}_{{{13}}} {-}{\text{ P}}_{{{32}}} {\text{r}}_{{{12}}} $$

4 $$ {\text{Direct }}\;{\text{effect }}\;{\text{of }}\;{\text{plant }}\;{\text{height }}\;{\text{on}}\;{\text{ the}}\;{\text{ flower }}\;{\text{number}}\;{\text{ per}}\;{\text{ plant}}:{\text{P}}_{{{32}}} = {\text{ r}}_{{{23}}} {-}{\text{ P}}_{{{31}}} {\text{r}}_{{{12}}} $$

5 $$ {\text{Direct }}\;{\text{effect }}\;{\text{of }}\;{\text{shoot }}\;{\text{weight }}\;{\text{per}}\;{\text{ plant }}\;{\text{on }}\;{\text{the}}\;{\text{ dry}}\;{\text{ flower }}\;{\text{yield}}:{\text{P}}_{{{41}}} = {\text{ r}}_{{{14}}} {-}{\text{ P}}_{{{42}}} {\text{r}}_{{{12}}} {-}{\text{ P}}_{{{43}}} {\text{r}}_{{{13}}} $$

6 $$ {\text{Direct}}\;{\text{ effect}}\;{\text{ of}}\;{\text{ plant}}\;{\text{ height }}\;{\text{on }}\;{\text{the}}\;{\text{ dry }}\;{\text{flower}}\;{\text{ yield}}:{\text{P}}_{{{42}}} = {\text{ r}}_{{{24}}} {-}{\text{ P}}_{{{41}}} {\text{r}}_{{{12}}} {-}{\text{ P}}_{{{43}}} {\text{r}}_{{{23}}} $$

7 $$ {\text{Direct}}\;{\text{ effect }}\;{\text{of }}\;{\text{flower }}\;{\text{number}}\;{\text{ per}}\;{\text{ plant}}\;{\text{ on}}\;{\text{ the}}\;{\text{ dry}}\;{\text{ flower }}\;{\text{yield}}:{\text{P}}_{{{43}}} = {\text{ r}}_{{{34}}} {-}{\text{ P}}_{{{41}}} {\text{r}}_{{{13}}} {-}{\text{ P}}_{{{42}}} {\text{r}}_{{{23}}} $$

Calculated values of the direct and indirect effects of each of growth characteristics and analyzed correlation coefficients between these traits and dry flower yield are presented in Table 6.

**Table 6:** Analyzed coefficients of correlation of the morphological characteristics affecting dry flower yield of *Echium amoenum* to direct and indirect effect.

Direct effect	
Shoot weight per plant (P_41_)	0.32
Plant height (P_42_)	0.27
Flower number per plant (P_43_)	0.24
**Indirect effect of shoot per plant via**	
Plant height (P_21_ × P_42_)	0.0594
Flower number per plant (P_31_ × P_43_)	0.0384
Plant height and Flower number per plant (P_21_ × P_32_ × P_43_)	0.0002
**Indirect effect of Plant height via**	
Flower number per plant (P_32_ × P_43_)	0.0012
Total direct and indirect effects of shoot yield per plant P_41_ + [(P_21_ × P_42_) + (P_31_ × P_43_) + (P_21_ × P_32_ × P_43_)]	0.418
Total direct and indirect effects of plant height P_42_ + (P_32_ × P_43_)	0.271
Residual effects (error)	0.07

The shoot weight per plant affected dry flower yield (P_31_ × P_43_ = 0.0384), but when its indirect effect through plant height and flower number per plant on dry flower yield simultaneously calculated, this effect was not significant (P_43_ × P_32_ × P_21_ = 0.0002).

**Figure 3:**
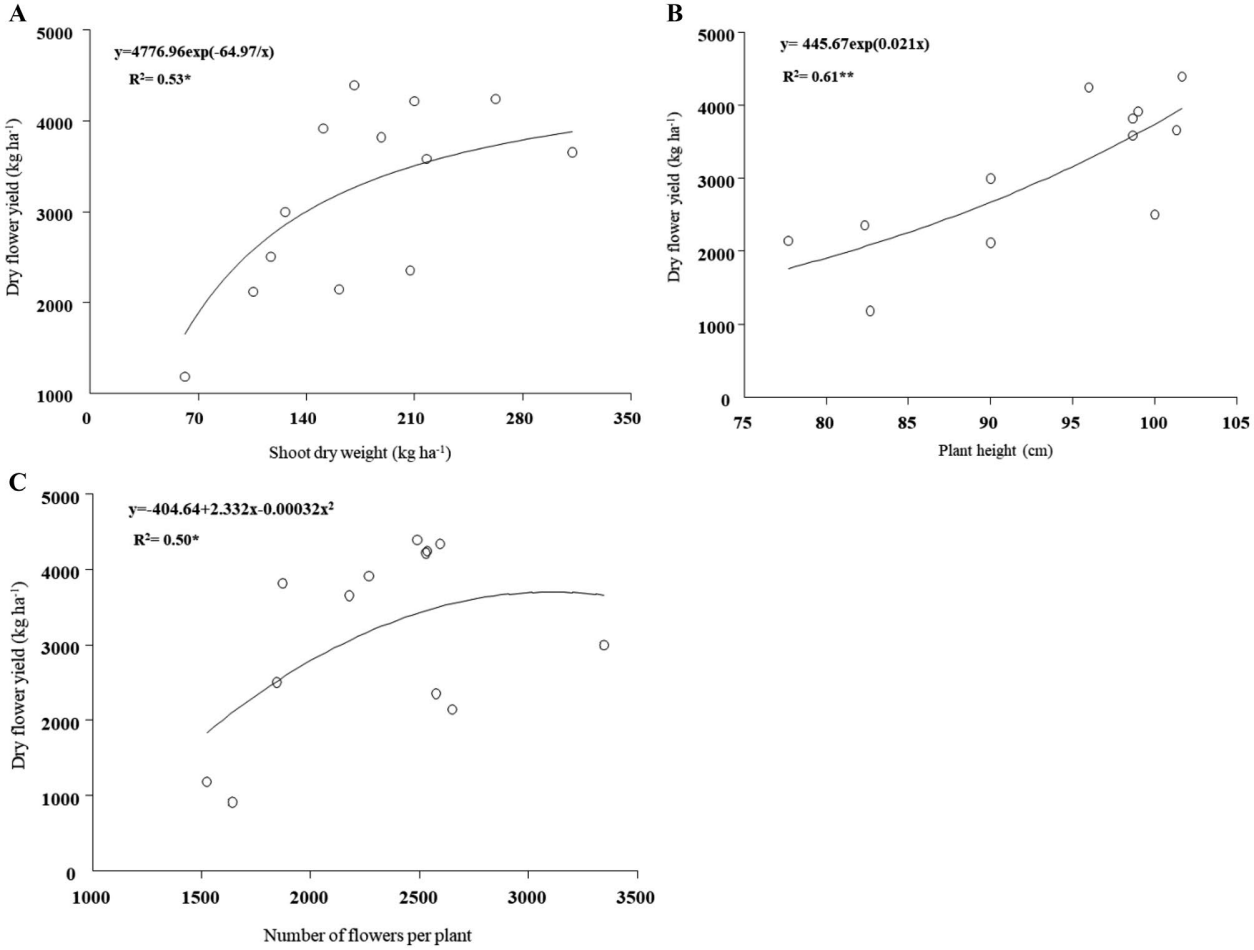
Regression functions of changes of dry flower yield of *Echium amoenum* against shoot dry weight (**A**), plant height (**B**) and flower number per plant (**C**).

Plant height indirectly affected dry flower yield through flower number per plant (P_32_ + P_43_ = 0.0012), as increased plant height led to increased flower number per plant and increased number of flowers per plant in turn resulted in dry flower yield (P_42_ + P_32_ + P_43_ = 0.271). This finding is confirmed by performed regressions between traits which correlations amongst those were already revealed (Table 5), for instance increasing each of the shoot weight per plant, plant height and flower number per plant resulted in increased dry flower yield (Fig. 3).

**Figure 4:**
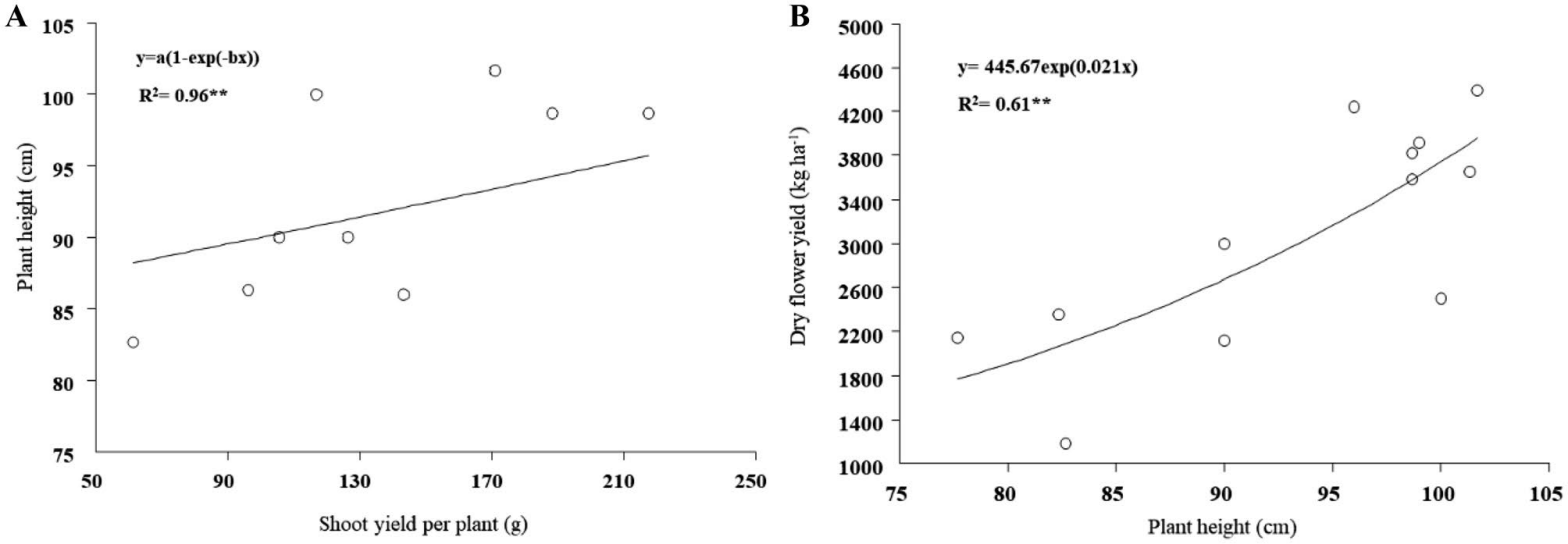
Indirect effect of shoot weight per plant on the dry flower yield of *Echium amoenum* mediated by plant height: (**A**) exponential relation between plant height and shoot yield, (**B**) exponential relation between plant height and dry flower yield.

As it shown in Fig. 4, there was a linear function between shoot weight per plant and plant height (Fig. 4A). On the other hand, increased plant height led to improved dry flower yield (Fig. 4B), so it is reasonable to expect that dry flower weight increases with increasing shoot yield.

## Discussion

### The effect of fertilizers and plant densiy

By reducing the number of plants per square meter, the positive effect of organic fertilizers on the growth characteristics of the plant was revealed obviously, probably due to more availability and synchrony of the nutrients in organic fertilizers to the plant needs. Soil organic matter content and biodiversity of living organisms in soil ecosystem, is known as the key of agro-ecosystem sustainability^36^. It seems that with decreasing plant density, plant access to growth resources such as light, water and nutrients is increased and resulted to improved growth characteristics of the plant in particular its height. The positive effects of organic fertilizers on the qualitative and quantitative characteristics of different plants have been emphasized in many studies^16,37,38^. Some researchers studied the effects of organic and chemical fertilizers on yield and essential oil percentage of basil (*Ocimum basilicum* L.) and reported that vermicompost-treated plants had higher plant height, leaf yield, shoot yield, fresh and dry yield than other treatments^39^^,^^40^.

It has been reported that the bio-organic fertilization caused an obvious increase in the microbial activity by enhancing acid phosphatase and dehydrogenase enzymes, bacterial count, and mycorrhizal colonization levels in maize rhizosphere as compared with the chemical fertilization^12^. It was found a significant and positive correlation (*R*^2^ = 0.52, 0.91 and 0.55) among maize grain yield and available N, P and K content, in soil. Higher levels of manure in soil usually lead to low pH^41^. On the other hand, P availability is highly dependent on soil pH^42^. It is widely accepted that the maximum P availability in the soil come through in pH range of 6.5–7.5 which meets the optimal needs of many plants. The soil temperature, CEC, and pH determine the availability of nutrients in the soil. Since pH levels control many bio-chemical processes that take place in the soil—specifically, plant nutrient availability—it is vital to maintain proper levels for plants to reach their full yield potential.

It was reported that organic fertilizers increased activity of microorganisms in soil^43^ and improved soil physiochemical and biological properties, increased nutrient storage capacity^44^ and slow release of nutrients. Nutrients improved the growth characteristics of the plant, including its height. The physiological mechanism involved in increasing plant height is probably as follows, when water and nutrients are sufficiently supplied to the plant, the accumulated water in the cells increases and is transmitted through the adjacent cells by turgidity, this mechanism is accompanied by changes in the ratio of plant hormones, including abscisic acid (ABA), gibberellin (GA), trans-zeatin-riboside (ZR), and indole acetic acid (IAA), eventually is resulted to the increased plant height^17^. Similar results were reported for guar plants inoculated by biofertilizers^13^. The effect of organic fertilizers on the qualitative and quantitative characteristics of medicinal plants of *Plantago ovate* Forsk., *Alyssum homolocarpum* L., *Lepidium perfoilatum* L. and *Lalementia iberica* L. were investigated and it was reported that cattle manure treatment produced the highest plant height compared with the other treatments, while the height of all plants was higher than the control due to application of vermicompost, coffee compost and mushroom compost^45^. The positive effects of vermicompost application on plant nutrition and growth was also reported for sorghum^8^.

It seems that the average plant density played the most role in increasing the flower number per plant. In low plant density, flower number per plant decreased compared with the average plant density probably due to excessive access to food and growth resources by the plant. In high plant density, this decrease was related to plant competition over water and nutrients and lack of efficient use of resources.

Organic fertilizers appear to be likely to increase the flower number per plant by supplying the plant with the micro nutrients^46^. Organic fertilizers might enhance number of flowers through improving soil microbial activities^16,47^, increasing water holding capacity^7^ and supplying more essential nutrients^46^, increased photosynthesis and plant dry matter^16,48^, which eventually led to increased flowering. It was reported that the bio-organic fertilization caused an obvious increase in the soil microbial activity by enhancing acid phosphatase and dehydrogenase enzymes, and bacterial count^12^. The effects of different organic and biological fertilizers on the safflower were studied and it was reported that vermicompost solely or combined with Nitroxin® and Nitrajin® biofertilizers improved the quality and quantity of the plant^49^. In a same study, it was reported that application of 10 t ha^−1^ vermicompost increased flower number, plant height, 1000-seed weight, biological yield and essential oil content of *Foeniculum vulgare* Mill^50^.

In high plant densities, it seems that intra-specific competition was increased and growth resources, particularly radiation, would not been adequately provided to the plant^19^, thus resulted in a decrease in dry flower yield. In a study, the effect of distance between planting rows (60, 70 and 80 cm) and within rows (25, 35 and 45 cm) on yield and yield components of *Satureja khuzistanica* Jamzad, was investigated. The results showed that the highest flower yield and canopy diameter were observed in 45 cm within row distance and density of 7 plant m^−2^ had the highest dry matter yield^51^.

The vermicompost probably played an important role in supplying the water needed for the plant^7^ because of its high moisture holding capacity, thereby producing more flower dry yield. It was reported that the positive effects of organic fertilizers including manure on soil water retention is provided enough amount of water for sustaining leaf growth, it has been also suggested that plants with more water content, contained more chlorophyll which in turn could be resulted in higher performance and yield^52^. Temperature of root region could affect nitrate absorption. Since organic matter such as cattle manure could affect the soil water holding capacity, thus, it could affect retaining and nutrients uptake (nitrogen in particular) too^53^. Cattle manure at low levels of plant density significantly increased dry flower yield probably through increased nitrogen release in soil^46^. Some studies have shown that the application of organic fertilizers reduce the salinity effects and increase the uptake of phosphorus and nitrogen thus improve the qualitative and quantitative characteristics of plants^16,54^. In a study, the effects of different levels of vermicompost (0, 5, 10, 15 and 20 t ha^−1^) on the qualitative and quantitative characteristics of German chamomile (*Matricaria chemmomilla*) were investigated. The highest dry and fresh flower yield, and the maximum plant height were obtained from vermicompost application of 20 t ha^−1^^55^. It was reported that soil inoculation with the optimized doses of biofertilizers saved about 25% of the chemical fertilizers required for the entire guar growth stages^13^.

Flower yield is a complex feature that is influenced by many physiological processes and its measurable performance would be revealed in phenological, morphological, and physiological traits^56^. Weak correlations between some traits appeared to be related to differences in the time of traits measured, as traits such as flower number per plant and flower weight per plant were measured during flowering, while the traits such as plant height, number of branches per plant and canopy diameter were evaluated at the end of the flowering period. Therefore, causality analysis was performed to accurately determine the contribution of each of the traits to improvement of dry flower yield.

Although the yield of most crops, particularly medicinal plants, has increased over the past decades, but the morphological and physiological processes underlying this increase of yield are not well identified^57^. Researches revealed the positively correlation between the physio morphological traits and yield of medicinal plants including *Mentha pulegium*, Peppermint (*Mentha piperita*) and *Thymus vulgaris*^58,59^. It has been reported that the application of 30 t ha^−1^ of manure under Eco environmental scenario caused high availability of N, P nutrients for plant, and improved crop productivity. Moreover, trapped and retained nutrients in manure matrix that is considered as an ecofriendly and low-cost input enrich soil fertility that improved the effectiveness of chemical fertilizer^24^. The application of compost and vermicompost fertilizers in density of 3 plants m^−2^ led to a 39 and 38% increase in total flavonoids compared to the control, respectively. The highest amount of total anthocyanin was obtained from the density of 5 plants m^−2^. Application of vermicompost and cattle manure increased seed oil by 10% and 13% and seed protein by 34% and 13%, respectively, compared to the control (The authors, In press).

If the origins of increased yield of medicinal plants are identified, paths to improve their actual potential by better crop management practice and effective nutrition supply may be identified^60^. In this study physio morphological traits affecting yield *Echium amoenum* were identified using multiple regression and causality analysis.

The coefficients of Eq. 1 show the relative impact of changes in each of the variables in the model on flower yield. For example, the change in flower yield was 0.32 units per unit of change in shoot yield per plant, while this change would be 0.27 per unit increase in plant height. In other words, the relative share of shoot yield per plant was about 16% higher than that of plant height, implying some important growth characteristics of borage such as producing numerous branches and flower formation at the end of them and finally the effect of these traits on flower yield. However, to better interpret these results, the unit of measurement for each variable should also be considered, which is why multiple regression was performed on standardized traits data. Thus, due to the effects of different treatments (different types of fertilizer and plant densities) in the above model, it is possible to quantitatively evaluate the response of borage based on the rate of increase or decrease of the variables affected by the treatments.

### Cause-and-effect analysis

Innovative results of cause-and-effect path analysis in this study indicate that shoot weight per plant had the most indirect effect on dry flower yield. The direct effect of shoot yield per plant on dry flower yield was more than direct effect of plant height via flower number per plant. Plant height had more direct effect on dry flower yield than flower number per plant (Table 6).

Shoot weight per plant affected dry flower yield indirectly in three ways:

(A) Indirect effect of shoot weight per plant through plant height (P_31_ + P_42_)
(B) Indirect effect of shoot weight per plant through flower number per plant (P_31_ + P_43_)
(C) Indirect effect of shoot weight per plant, by plant height and number by flowers per plant (P_21_ + P_32_ + P_43_)

As it shown in Table 6, shoot weight per plant had the most indirect effect on dry flower yield (0.0594), which resulted in increased dry flower yield mediated through plant height. It was reported that the yield of flowering branches of *Camphorosma monspeliaca* L. was positively correlated and affected by shoot yield. Also, the number of tillers that had the most direct effect on the yield of flowering branches was also indirectly affected by plant height^61^.

The results (Table 6) showed that the direct effect of plant height on dry flower yield (P_42_ = 0.27) was more than the indirect effect through flower number per plant (P_43_ × P_32_ = 0.0012). In a study, investigation of morphological characteristics affecting yield of medicinal plant revealed that improvement of plant height and branch number per plant increased flowering branches yield^61^. The results of causality analysis indicated that flower number per plant had only a positive direct effect on dry flower yield. From a physiological point of view, the flower number in plants such as borage is the last component of yield and cannot transmit yield fluctuations (in this case, flowers) to another component, thus the causality analysis performed is fully consistent with the physiological bases. Comparison of morphological traits affecting dry flower yield showed that shoot weight per plant affected dry flower yield more than the other traits, as the total direct and indirect effects of shoot yield per plant was more than the other traits. Considering the paths coefficient affecting dry flower yield (Fig. 2), it seems that management practices and treatments that would increase shoot yield per plant would lead to improved yield of *Echium amoenum*. Results of a study on sunflower (*Helianthus annus* L.) showed that there were positive correlations between biological yield, shoot yield and grain yield^62^. In another study, shoot yield, plant height and number of grains per plant were identified as the most influential traits on the yield of *Trigonella foenum-graecum* L^63^. Some researchers reported that shoot yield, particularly umbrellas dry weight and 1000-seed weight of *Coriandrum sativum* L. were the most important traits affecting the yield of this medicinal plant^64^. Guler et al.^26^ determined negative and significant relationship between 100-seed weight and seed yield of chickpea using path coefficient analysis.

**Figure 5:**
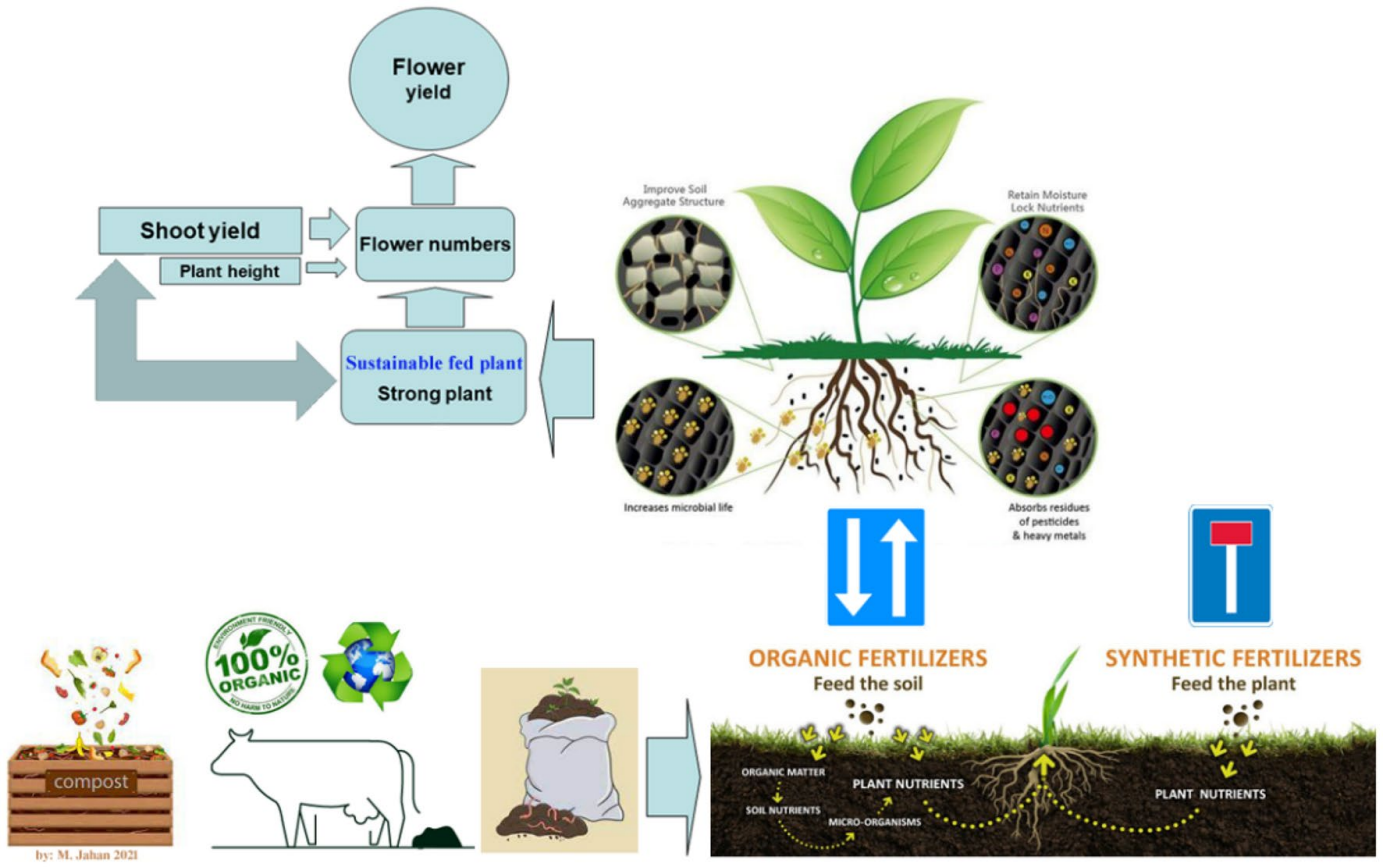
Organic fertilizers versus chemical fertilizers, and the related processes affect the flower yield of Echium amoenum through the explored causal paths.

In brief and generally, the benefits of application of organic fertilizers and the path that these fertilizers and dependent processes affect the final yield of the plant shown in Fig. 5 schematically.

## Conclusions

The results showed that cultivation of borage with density of 5 plants m^−2^ and application of compost resulted to the highest flower yield. There was a significant positive correlation between dry flower yield and all studied growth characteristics except seed yield and canopy diameter. According to the results of multiple regression, shoot yield per plant, plant height and flower number per plant were identified as the main factors affecting dry flower yield, although the relative proportion of plant height compared with shoot weight per plant and flower number was higher by 16 and 25%, respectively. Causality analysis revealed that shoot weight per plant had the most direct effect on dry flower yield, while this trait through three paths (1-plant height, 2-flower number per plant, 3-Plant height) had an indirect effect on dry flower yield. The causality analysis also identified that shoot weight per plant seems affected dry flower yield through plant height, along with increasing shoot weight per plant, plant height was increased which in turn improved dry flower yield.

Conclusively, this study suggests that optimum production of *Echium amoenum* with application of ecological inputs along with effective agronomical managements of the causal paths of flower yield forming, including increase in shoot yield and plant height could be achieved through an ecological cropping system. Moreover, achieving more yield from organic fertilizers application than chemical fertilizer in this study, promises agrochemicals free and healthy production of this medicinal plant could be achieved from low input cropping systems or marginal farms using ecological inputs.

## Data Availability

All used and created data are available on demand.
